# 基于二代基因测序技术对RUNX1-RUNX1T1阳性急性髓系白血病的临床特征及预后分析

**DOI:** 10.3760/cma.j.issn.0253-2727.2023.10.010

**Published:** 2023-10

**Authors:** 亚丽 王, 素君 高, 龙 苏, 亚娟 刘, 云蔚 张, 雅哲 杜

**Affiliations:** 吉林大学第一医院肿瘤中心血液科，长春 130021 Cancer Center, the First Hospital, Jilin University, Changchun 130021, China

急性髓系白血病（AML）是一种常见的血液系统恶性疾病，而RUNX1-RUNX1T1融合基因阳性AML是最常见的亚型之一[1]–[2]，它具有独特的形态学和免疫表型，属于核心结合因子阳性白血病的一种[3]。虽然美国国家综合癌症网络指南将此类AML归为低危组[4]，仅用化疗，尤其是大剂量阿糖胞苷为基础的巩固治疗方案，长期存活率可达60％，但疾病复发仍然是治疗失败的最重要的原因，发生率高达30％～40％[5]–[7]。在本研究中，我们旨在通过分析我中心RUNX1-RUNX1T1阳性AML患者的临床特征、实验室结果及预后，评价基于二代测序技术检测下的RUNX-RUNX1阳性AML预后的影响因素，以期更好地协助诊治，指导临床决策。

## 病例与方法

1. 病例：回顾性分析2016年1月至2020年12月就诊于我中心的113例初治 RUNX1-RUNX1T1阳性AML患者。患者年龄均大于14岁。患者初治骨髓标本均进行细胞形态学、免疫学、细胞遗传学及分子生物学（二代测序）等检测，诊断符合文献[4]标准。

2. 治疗：诱导化疗采用DA（柔红霉素+阿糖胞苷）、IA（去甲氧柔红霉素+阿糖胞苷）、DCAG（地西他滨+阿克拉霉素+阿糖胞苷+G-CSF）或MAE（米托蒽醌+阿糖胞苷+依托泊苷）方案。完全缓解后采用中大剂量阿糖胞苷为主的巩固化疗，不适合的患者接受HAA（高三尖杉酯碱+阿糖胞苷+阿柔比星）、AA方案或其他治疗。并给予腰椎穿刺+三联鞘内注射（甲氨蝶呤、阿糖胞苷、地塞米松）预防中枢神经系统白血病，符合条件的患者行造血干细胞移植（HSCT）。

3. 疗效判定：参照文献[8]疗效分为形态学完全缓解（CR）、形态学完全缓解伴血细胞计数未完全恢复（CRi）、部分缓解（PR）、治疗失败及复发。总生存（OS）时间定义为自确诊日至患者死亡或随访截止日期。无复发生存（RFS）时间定义为CR至白血病复发或 CR期内死亡的时间。早期死亡定义为生存期≤4周。

4. 随访：随访方式包括电话、门诊记录、医院病例登记系统等。自患者确诊之日起进行随访，随访截至2022年1月。

5. 统计学处理：本研究采用SPSS 25软件进行数据统计分析。计量资料组间比较采用独立样本*t*检验（正态分布且整体方差齐）或非参数检验（非正态分布或整体方差齐）。计数资料组间比较采用卡方检验。多因素分析用Logisitic回归。生存分析采用Kaplan-Meier法绘制生存曲线，组间比较采用Log秩和检验。单因素分析中*P*<0.10的因素纳入Cox 回归模型进行多因素分析。*P*<0.05为差异有统计学意义。

## 结果

1. 一般临床特征：本组113例AML患者中男55例，女58例，中位年龄46（14～73）岁，初诊中位WBC为7.4（1.0～93.0）×10^9^/L，骨髓中位原始细胞比例为57.0％（8.0％～98.5％）。101例（89.38％）患者成功获得染色体核型分析结果，其中71例有附加染色体异常，主要为性染色体缺失（53.5％，54/101）。

2. 二代测序结果：113例患者中111例行二代测序基因突变筛查，99例（89.2％）检出至少1个基因突变，共检出55种突变，其中KIT突变44例（39.6％）（KIT D816 21例，18.9％），NRAS突变22例（19.8％），FLT3突变17例（15.3％）（TKD 12例，ITD 5例），JAK2突变10例（9.0％），KRAS突变10例（9.0％），ASXL1突变9例（8.1％），CSF3R突变9例（8.1％），RAD21突变8例（7.2％），KDM6A突变8例（7.2％），EZH2突变8例（7.2％），ASXL2突变7例（6.3％）。

按功能分类，酪氨酸激酶信号通路相关基因突变最常见（65.8％，73/111），其次为表观遗传学调控（25.2％，28/111）与DNA甲基化相关突变（16.2％，18/111），其他分类基因突变发生率低于10％，包括黏连蛋白复合物相关（9.0％，10/111）、肿瘤抑制基因相关（9.0％，10/111）、转录调控相关（7.2％，8/111）、剪接体相关基因突变（1.8％，2/111）。

111例患者中12例（10.8％）无合并突变，合并1个和2个突变的患者最多，均为33例（29.7％），合并3个和4个突变者均为11例（9.9％），合并5、6、7、8个突变者分别为6例（5.4％）、3例（2.7％）、1例（0.9％）、1例（0.9％）。合并超过2个突变的患者占59.5％。这些检测到的基因突变中，最常见的两个突变的组合为KIT/NRAS（8.1％，9/111）。

3. 生存分析：排除未接受治疗或失访、未规律治疗患者，55例患者在达到CR或CRi之后接受了2～9个疗程巩固治疗，9例患者行HSCT，共64例纳入生存分析。病例中位随访时间25（6～72）个月，中位OS时间52个月，中位RFS时间43个月，存活38例（59.4％），死亡21例（32.8％），复发15例（23.4％），10例复发后死亡，2年OS率为79.6％，RFS率为70.6％。

对可能影响患者预后的一般临床特征，如患者性别、年龄、初诊时WBC、骨髓及外周血原始细胞、流式细胞术微小残留病（MRD）、RUNX1-RUNX1T1融合基因定量、染色体核型、第1疗程疗效进行单因素分析，结果显示初诊时WBC>25×10^9^/L患者共10例，OS期明显短于WBC≤25×10^9^/L患者（*P*＝0.034），RFS期差异无统计学意义（*P*＝0.234）。而其他的一般临床特征对患者预后无显著影响（*P*值均>0.05）。

对发生频率较高的基因突变进行单因素分析，结果显示基因突变中，合并KIT突变的患者共19例，中位RFS期明显短于KIT突变阴性患者（*P*＝0.022），中位OS期差异无统计学意义（*P*＝0.140），而KIT D816突变的患者共9例，其中位OS及RFS期明显缩短（*P*＝0.007，*P*＝0.003）。合并FLT3突变的患者共11例，同样OS及RFS较差（*P*＝0.002，*P*＝0.002），而合并FLT3-ITD突变患者太少无法分析。合并NRAS基因突变对预后无明显影响。

MRD分为流式细胞术及RUNX1-RUNX1T1融合基因定量检测两种，流式细胞术MRD阴性定义为下降至<0.01％，融合基因MRD阴性定义为RUNX1-RUNX1T1下降>3个log值。本研究中，2个疗程后流式细胞术MRD转阴的患者共50例，该组患者中位OS期及RFS期明显长于未转阴患者（*P*＝0.007，*P*＝0.047），而RUNX1-RUNX1T1 融合基因4个疗程后转阴的患者共39例，与未转阴患者比较，RFS（*P*＝0.080）及OS（*P*＝0.230）差异均无统计学意义，但融合基因阳性患者有预后不良趋势。

将单因素分析中*P*<0.10的因素代入多因素分析，因融合基因定量与流式细胞术均可检测MRD，本研究中选择流式细胞术MRD阳性代入。初诊WBC>25×10^9^/L、KIT D816突变、2个疗程后流式细胞术MRD阳性为影响OS的独立危险因素，KIT D816突变、FLT3突变、2个疗程后流式细胞术MRD阳性为影响RFS的独立危险因素（表1）。

**表1:** RUNX1-RUNX1T1阳性急性髓系白血病患者总生存和无复发生存的多因素分析

影响因素	总生存存		无复发生存	
	*HR*(95%*CI*)	*P*值	*HR*(95%*CI*)	*P*值
初诊WBC>25×10^9^/L	0.326（0.118~0.897）	0.030		
KIT D816阳性	0.216（0.073~0.645）	0.006	0.309（0.120~0.795）	0.015
NRAS阳性	0.673（0.245~1.847）	0.442		
FLT3阳性	0.483（0.174~1.342）	0.163	0.363（0.152~0.864）	0.022
2个疗程后流式细胞术MRD阳性	0.215（0.072~0.638）	0.006	0.385（0.153~0.970）	0.043

注 MRD：微小残留病

## 讨论

合并RUNX1-RUNX1T1融合基因的白血病通常属于低危组，但多个相关研究表明其复发率很高。因而，深入研究此类患者预后再分层对优化临床患者管理、改善患者疗效具有重要意义。

RUNX1-RUNX1T1融合基因不能单独诱发白血病，而是与继发性突变协同诱发，在白血病的发生发展过程中呈现“双打击模型”[9]–[11]。本研究显示高达89.2％的患者在诊断时出现1～8个基因突变，其中最常见的突变是KIT突变，发生率为39.6％，与中外文献报道大致相同[7],[12]–[13]，多项研究表明伴随KIT基因突变的RUNX1- RUNX1T1融合基因阳性AML患者OS及RFS均较差[5],[12]–[15]，本研究也得出了相同的结果，而且KIT基因突变中D816突变对预后影响较大。这可能与 D816突变具有更强的促进细胞增殖和抗凋亡作用有关[16]。

FLT3基因突变分为近膜区的内部串联重复序列（ITD）和酪氨酸激酶结构域活化环内的点突变（TKD），在成人AML及RUNX1- RUNX1T1融合基因阳性AML患者中发生率大致相等，均为20％左右[14],[17]–[18]，本研究中其发生率为15.3％，与国内外研究一致。而FLT3-TKD突变发生率比FLT3-ITD高，与已知结果不同[17]，这可能与地域或样本量较少有关。FLT3基因突变通常与较差预后相关[19]，而导致这样结果的主要为FLT3-ITD突变[7],[14]，本研究中进入预后分析的FLT3阳性患者OS及RFS较差，其中FLT3-ITD阳性患者OS期与RFS期明显缩短。

初诊时WBC也是影响预后的又一因素，多项研究表明WBC越高，预后越差[5],[14]。本研究中患者初治时高白细胞的患者OS较差。

有研究显示RUNX1-RUNX1T1表达水平是RUNX1- RUNX1T1融合基因阳性AML的独立预后因素[20]。英国MRC AML-15临床试验结果提示1疗程化疗后RUNX1-RUNX1T1转录本下降大于3个log值提示预后良好[21]。而国内AML05多中心临床试验结果显示，中大剂量阿糖胞苷巩固治疗2个疗程后，如果RUNX1-RUNX1T1转录本下降超过3个log值且持续半年以上定义为低危组，否则为高危组[22]。本研究中4个疗程化疗后转录本下降超过3个log值的患者OS及RFS无统计学意义，但生存曲线可见MRD阳性患者有预后不良趋势，这可能与移植延长了患者生存、样本量不足以及随访时间较短有关。另有研究认为使用多参数流式细胞术检测的MRD的存在与较差的OS和RFS显著相关[19],[23]，本研究中，2个疗程化疗后流式细胞术MRD阳性是影响OS及RFS的独立预后不良因素，因此流式细胞术MRD监测是早期识别预后不良患者的重要因素，为指导治疗决策提供帮助。我们的研究结果表明，在临床实践中，两种MRD分析在优化风险分层方面具有互补作用。

综上所述，KIT-D816、FLT3、WBC、2个疗程后流式细胞术MRD均为RUNX1-RUNX1T1融合基因阳性AML患者预后的影响因素。
